# Single-cell multi-omics and nursing follow-up prognostic modeling reveal SLFN4-mediated neutrophil dysregulation in traumatic brain injury

**DOI:** 10.3389/fimmu.2025.1669800

**Published:** 2025-09-23

**Authors:** Wenping Tang, Yang Wang, Fu Zhao, Yang Hong, Lina Wang, Wanyan Xu, Fangfang Ding, Tingting Shi, Jing Ke, Li Zhuang, Yuan Wang, Hongling Jia, Huabao Cai, Xiumei Zhang, Wei Duan

**Affiliations:** ^1^ Department of Neurosurgery, The First Affiliated Hospital of Anhui Medical University, Anhui Medical University, Hefei, China; ^2^ Department of Neurosurgery, Shanghai Ninth People’s Hospital, School of Medicine, Shanghai Jiao Tong University, Shanghai, China; ^3^ School of Traditional Chinese Medicine, Jinan University, Guangzhou, China; ^4^ College of First Clinical Medicine, Shandong University of Traditional Chinese Medicine, Jinan, China; ^5^ Center for Scientific Research of Anhui Medical University, Anhui Medical University, Hefei, Anhui, China; ^6^ Institute of Health and Medicine, Hefei Comprehensive National Science Center, Hefei Economic and Technological Development Zone, Hefei, Anhui, China; ^7^ Department of Radiology, The First Affiliated Hospital of Anhui Medical University, Anhui Medical University, Hefei, China

**Keywords:** traumatic brain injury, nursing follow-up prognostic model, SLFN4-positive neutrophils, immune heterogeneity, precision nursing care, multi-omics integration

## Abstract

**Background:**

Traumatic brain injury (TBI) is increasingly recognized as a systemic inflammatory disorder, with neutrophils playing a critical role in secondary injury. However, the phenotypic heterogeneity and clinical significance of neutrophil subsets in the early TBI immune landscape remain unclear, limiting their utility in nursing prognostic assessment and individualized care planning.

**Methods:**

We performed an integrated multi-omics analysis—combining single-cell RNA sequencing (scRNA-seq), bulk transcriptomics, and proteomics—to dissect neutrophil diversity post-TBI. A distinct SLFN4^+^ neutrophil population was identified and further validated through *in vitro* functional assays and serum profiling in a TBI patient cohort. Clinical correlations and nursing stratification models were constructed to evaluate prognostic relevance.

**Results:**

At 24 hours post-injury, scRNA-seq revealed four neutrophil clusters in mouse brains. Among these, the SLFN4^+^ subset exhibited N1-like polarization, pro-inflammatory activation, and metabolic rewiring favoring glycolysis and oxidative phosphorylation. Regulon and pseudotime analyses highlighted its transitional regulatory potential. SLFN4^+^ neutrophils actively engaged in TNF and CCL-mediated communication with monocytes/macrophages. Functionally, silencing SLFN4 or STAT2 enhanced neutrophil proliferation and reduced inflammatory cytokine secretion. Clinically, elevated serum SLFN4 levels in TBI patients were associated with poor neurological outcomes and, when incorporated into a composite nursing risk model, significantly improved early prognostic accuracy.

**Conclusions:**

SLFN4^+^ neutrophils represent a key inflammatory effector population contributing to early immune dysregulation after TBI. Beyond mechanistic insights, SLFN4 serves as a promising serum biomarker to enhance clinical decision-making and nursing risk stratification. These findings support the integration of immunological biomarkers into precision nursing frameworks to guide early interventions and improve neurorehabilitation outcomes.

## 1 Introduction

Traumatic brain injury (TBI) is a critical public health concern and a leading cause of disability and mortality, particularly among young and middle-aged adults worldwide. While initial mechanical trauma causes direct neurological damage, the ensuing secondary injury cascade—driven largely by immune-inflammatory responses—plays a decisive role in neurological deterioration, delayed recovery, and long-term functional outcomes (1, 2). These secondary responses often complicate clinical care and represent important therapeutic and prognostic targets in both acute management and long-term rehabilitation settings (3).

Among the earliest responders to TBI-induced inflammation are neutrophils, which infiltrate the damaged brain parenchyma within hours post-injury. Although neutrophils are essential for host defense and tissue decontamination, their overactivation can lead to the excessive release of reactive oxygen species, proteases, and pro-inflammatory cytokines, thereby exacerbating blood-brain barrier disruption, neuronal injury, and cerebral edema (4–10). From a clinical perspective, persistent neutrophilic inflammation has been linked to poor neurological outcomes and prolonged intensive care needs. Understanding the heterogeneity of neutrophil phenotypes in TBI and their molecular regulators is therefore crucial for guiding individualized treatment strategies and early risk stratification in neurosurgical and neurocritical care settings.

Accurate prognostic evaluation is central to nursing care planning and post-trauma rehabilitation in patients with traumatic brain injury (TBI). Conventional nursing risk stratification models often rely on clinical indicators such as Glasgow Coma Scale (GCS) score, imaging findings, and vital signs. While these parameters offer rapid bedside assessments, they may not fully capture the underlying molecular heterogeneity and immune-inflammatory dynamics that drive patient deterioration or recovery. This disconnect limits the predictive accuracy of current models and hampers early personalized interventions. Recent advances in transcriptomics, especially single-cell RNA sequencing (scRNA-seq), have enabled unprecedented resolution in characterizing immune cell subpopulations and their functional trajectories following TBI. These technologies provide valuable insights into systemic inflammatory patterns that can complement conventional nursing assessment tools. By integrating such multi-omics findings into nursing prognostic models, we can bridge the gap between bench and bedside, enabling stratified care strategies, enhanced early-warning systems, and more precise rehabilitation planning. Importantly, this approach empowers nursing professionals to engage with precision health data and translate complex molecular signatures into actionable care decisions.

The Schlafen (SLFN) family comprises interferon-regulated proteins with diverse roles in immune cell differentiation, proliferation, and activation. Among them, Schlafen family member 4 (SLFN4) has emerged as a modulator of myeloid cell function and inflammatory polarization (11, 12). However, the relevance of SLFN4 in the context of neuroinflammation (13) and its specific involvement in neutrophil-mediated responses after TBI remains largely unexplored (14, 15). Identifying SLFN4-expressing neutrophils may offer a new immunological biomarker for early risk evaluation and personalized nursing interventions.

Advances in high-throughput sequencing and proteomic technologies have enabled the dissection of immune cell states at single-cell resolution (16). Integration of single-cell RNA sequencing (scRNA-seq), bulk transcriptomic profiling, and proteomic validation offers a powerful framework to uncover novel immune signatures and link them to functional and clinical outcomes (12, 17). This multi-omics strategy is especially valuable in dissecting the complexity of the post-TBI immune landscape (18).

In this study, we utilized an integrative multi-omics strategy to characterize neutrophil heterogeneity in early-stage TBI. We identified a distinct SLFN4^+^ neutrophil subset exhibiting N1-like pro-inflammatory signatures and metabolic remodeling. Importantly, SLFN4^+^ neutrophil abundance was significantly associated with Glasgow Outcome Scale–Extended (GOSE) scores and Barthel Index scores during longitudinal nursing follow-up, underscoring its prognostic value in patient recovery trajectories. Our findings not only reveal mechanistic insights into TBI-associated neuroinflammation but also propose SLFN4 as a clinically actionable biomarker to guide risk stratification, patient monitoring, and targeted nursing interventions (19).

## 2 Methods

### 2.1 Single-cell sequencing data acquisition

Single-cell transcriptomic data relevant to TBI were obtained from the Gene Expression Omnibus (GEO) under accession number GSE269748 (https://www.ncbi.nlm.nih.gov/geo/). This dataset contains single-cell RNA sequencing profiles of both immune and non-immune cell populations isolated from the brains of mice subjected to controlled cortical impact (CCI) and sham-operated controls, all collected at 24 hours post-injury (18, 20). The dataset includes samples from four TBI mice and four sham-treated mice. As the data are publicly accessible, no additional ethical approvals were required for their use. To ensure reproducibility and analytical rigor, information regarding sequencing protocols, library construction, and metadata were retrieved from the GEO database entry and its corresponding published reference (21).

### 2.2 Single-cell data processing and visualization

Raw single-cell RNA sequencing data were analyzed in R (version 4.3.3) using the Seurat package (version 4.4.0). Doublet artifacts were identified and excluded using the DoubletFinder algorithm (v2.0.3). Quality control thresholds were applied to retain high-confidence cells, specifically those with 300–6,000 detected genes, 500–100,000 unique molecular identifiers (UMIs), mitochondrial gene content below 25%, and erythroid/hemoglobin gene expression under 5%. Normalization was conducted via the NormalizeData function, and the top 2,000 most variable genes were selected using FindVariableFeatures. The dataset was subsequently scaled using ScaleData, followed by principal component analysis (PCA) for dimensionality reduction. Batch effects across samples were mitigated using the Harmony integration algorithm (v0.2.1), and the first 30 principal components were employed for clustering and Uniform Manifold Approximation and Projection (UMAP) visualization.

Cell type classification was performed using canonical marker genes in reference to the CellMarker database. Neutrophils were computationally subsetted from the broader dataset, re-clustered independently, and further subdivided into distinct subpopulations based on established subtype-specific markers (22–24).

### 2.3 Cell preference assessment and functional enrichment analysis

To evaluate cell-type-specific enrichment between TBI and control conditions, odds ratio (OR) analysis was employed to compare the relative abundance of each cellular population. Log-transformed frequencies were utilized to infer enrichment patterns and tissue distribution trends (25, 26).

Cluster-specific and subcluster-specific differentially expressed genes (DEGs) were identified using the FindAllMarkers function in Seurat, based on the Wilcoxon rank-sum test. DEGs were filtered using thresholds of |log_2_ fold change| > 0.25 and adjusted p-value < 0.05 (27). Gene Ontology (GO) and Kyoto Encyclopedia of Genes and Genomes (KEGG) pathway enrichment analyses were performed using the clusterProfiler package (v4.8.2) and the Single-Cell Portal (SCP). Gene Set Enrichment Analysis (GSEA) was further applied to elucidate functional pathways associated with DEGs (28–31).

To evaluate gene set activity at the single-cell level, the AUCell framework was utilized. Cell-wise enrichment scores were calculated via AUCell_buildRankings and AUCell_calcAUC functions. Gene sets relevant to stemness, inflammation, and immune regulation were curated from the Molecular Signatures Database (MSigDB) and prior peer-reviewed publications (27, 32, 33).

### 2.4 Cell lineage trajectory analysis

To explore neutrophil developmental trajectories following TBI, we employed multiple complementary pseudotime and lineage reconstruction algorithms. Transcriptional plasticity and differentiation potential were initially assessed using CytoTRACE (v0.3.3), which estimates cellular stemness based on gene expression entropy and transcriptomic diversity.

Monocle 2 (v2.24.0) was subsequently utilized to perform pseudotemporal ordering of cells. Highly variable genes were selected to construct differentiation trajectories through the reduceDimension and orderCells functions. This approach allowed inference of temporal progression along a continuous developmental path.

To further delineate lineage relationships, Slingshot (v2.6.0) was applied using principal component data derived from either Seurat or Harmony integration as input. Lineage topologies and branching structures were reconstructed using the getLineages and getCurves functions. These trajectories were visualized in the UMAP embedding to highlight bifurcation points and dynamic gene expression changes.

### 2.5 Gene set scoring

To quantify the activation of predefined gene sets at the single-cell level, we implemented the AUCell algorithm through the irGSEA R package. For each cell, gene expression profiles were ranked, and the enrichment of target gene sets was evaluated by calculating the area under the curve (AUC) scores.

The curated gene sets encompassed biological pathways associated with stemness, inflammatory signaling, immune activation, and neutrophil-specific functions. These were sourced from established databases including MSigDB and Reactome.

Enrichment scores were computed using the AUCell_buildRankings and AUCell_calcAUC functions. Visualization of score distributions was performed using UMAP projection and violin plots. Thresholds for positive enrichment were defined based on internal negative controls or bimodal distribution patterns.

### 2.6 Intercellular communication network construction

To elucidate patterns of intercellular communication within the TBI-affected brain microenvironment, we employed the CellChat R package (version 1.6.1), which infers signaling interactions based on curated ligand–receptor pairings.

Preprocessed single-cell RNA-seq expression matrices and cell identity labels derived from Seurat clustering were input into CellChat using the createCellChat function. Interaction databases were referenced from CellChatDB (https://github.com/sqjin/CellChat) for ligand–receptor annotations.

Signaling probability scores were computed using the computeCommunProb function, applying a permutation-based significance cutoff of P < 0.05. Global intercellular communication patterns were summarized using computeCommunProbPathway and aggregateNet.

Comparative analyses between cell types or experimental conditions (e.g., sham vs. TBI) were performed using netVisual_diffInteraction and netAnalysis_signalingRole. Communication roles were further dissected into incoming and outgoing signals based on computed centrality scores.

Key signaling pathways—such as TNF, CXCL, interferons, and PDGF—were visualized through circle plots and hierarchical clustering of pathway-specific interactions. This network analysis delineated the directional and context-specific features of immune-mediated communication in the injured brain (34).

### 2.7 Gene regulatory network construction based on SCENIC

Gene regulatory networks at single-cell resolution were constructed using the SCENIC pipeline, executed via pySCENIC (v0.10.0) in a Python 3.7 environment. The analysis proceeded in three key steps: First, gene co-expression modules were inferred using the GRNBoost2 algorithm, which calculates gene–gene correlations based on log-normalized expression matrices to predict preliminary transcription factor (TF)–target interactions. Next, these interactions were refined through cis-regulatory motif enrichment analysis utilizing the cisTarget database; only those TF–target pairs with enriched binding motifs within ±10 kb of transcription start sites were retained to define high-confidence regulons. Finally, the AUCell algorithm was applied to quantify regulon activity at the single-cell level by calculating area under the curve (AUC) scores, producing a regulon activity matrix across all cells. Visualization via UMAP and heatmaps enabled comparison of regulon dynamics among neutrophil subpopulations and other immune cell types in the TBI brain. Key transcriptional regulators implicated in inflammation and cell state transitions, including Irf7, Stat1, Batf, and Cebpb, were identified as central drivers of neutrophil functional heterogeneity (35).

### 2.8 Clinical cohort follow-up and prognostic stratification

To investigate the prognostic value of SLFN4 in TBI, we conducted a prospective observational study involving adult patients (≥18 years) diagnosed with moderate-to-severe traumatic brain injury who were hospitalized at the First Affiliated Hospital of Anhui Medical University between January 2023 and December 2024. The studies involving human participants were reviewed and approved by the Ethics Committee of The First Affiliated Hospital of Anhui Medical University (Reference Number: Quick–PJ 2023-14-88, and all procedures were conducted in accordance with the Declaration of Helsinki and relevant national guidelines. The study adhered to the principles outlined in the Declaration of Helsinki, with informed consent obtained from all participants. During hospitalization, structured face-to-face nursing assessments were conducted on admission day 1, day 3, and day 7 to evaluate neurological function, systemic inflammation, and early recovery status. After discharge, standardized telephone follow-ups were performed at 1, 3, and 6 months to continuously monitor patients’ functional outcomes, inflammatory status, and complication burden. Follow-up assessments were conducted by trained neurosurgical nurses using validated clinical instruments: Glasgow Coma Scale (GCS) and Extended Glasgow Outcome Scale (GOSE) for neurological function; neutrophil-to-lymphocyte ratio (NLR), C-reactive protein (CRP), and serum SLFN4 levels for inflammatory status; Barthel Index and EQ-5D for functional recovery; and complication tracking for fever, seizures, and infections. Clinical data were recorded in an encrypted electronic case report form (CRF) and verified independently by two researchers. For SLFN4 quantification, peripheral venous blood (5 mL) was collected on post-admission day 1, and at 1 and 3 months. After centrifugation (3,000 × g, 10 minutes), serum was stored at −80 °C and analyzed in duplicate using a commercially available ELISA kit (Cat# SLFN4-HU-ELISA; Cloud-Clone Corp., Wuhan, China). The assay exhibited intra- and inter-assay coefficients of variation below 10%. To stratify patient risk, a composite prognostic score was developed by integrating standardized values (z-scores) of SLFN4, NLR, GCS, GOSE, Barthel Index, and complication burden. Weighting coefficients (β_i_) were derived from multivariate Cox regression, and the cumulative risk score was calculated as $Risk\;Score=\sum \left(\beta_{i}\;\times \;Z_{i}\right)$ . Patients were classified into low-, intermediate-, and high-risk groups based on tertile thresholds. Prognostic differences were evaluated using Kaplan–Meier survival curves and log-rank testing, while model discriminative performance was assessed via receiver operating characteristic (ROC) analysis and area under the curve (AUC) metrics.

### 2.9 Cell culture, induced differentiation, and shRNA transduction

Human promyelocytic leukemia cell lines HL-60 and NB4 were obtained from the Cell Bank of the Chinese Academy of Sciences (Shanghai, China) and maintained in RPMI-1640 medium (Gibco, Cat#11875-093) supplemented with 10% fetal bovine serum (FBS; Gibco, Cat#10099-141) and 1% penicillin–streptomycin (Gibco, Cat#15140-122). Cells were cultured under standard conditions at 37 °C in a humidified incubator with 5% CO_2_.

To induce neutrophil-like differentiation, cells were treated with 1.3% dimethyl sulfoxide (DMSO; Sigma, Cat#D2650) for a duration of 5 days. Successful differentiation was validated through the upregulation of CD11b surface expression as assessed by flow cytometry, and by the observation of morphological changes characteristic of neutrophils using Wright–Giemsa staining.

Following differentiation, cells were transduced with lentiviral vectors encoding short hairpin RNAs (shRNAs) (detailed in **Supplementary Table 1**) targeting either Slfn4 or STAT2 (GenePharma, Suzhou, China) in the presence of 8 μg/mL polybrene (Sigma, Cat#H9268) to enhance transduction efficiency. Two independent shRNA sequences per gene target were used to ensure knockdown specificity. Stable integration was achieved by selection with 2 μg/mL puromycin (Sigma, Cat#P9620) for 5 consecutive days prior to downstream functional assays.

### 2.10 qRT-PCR analysis

Total RNA was extracted from DMSO-differentiated HL-60 and NB4 cells using TRIzol™ reagent (Invitrogen, Cat#15596026). RNA purity and concentration were assessed using a NanoDrop 2000 spectrophotometer (Thermo Fisher Scientific). One microgram of RNA was reverse-transcribed into complementary DNA (cDNA) using the PrimeScript™ RT reagent kit with gDNA Eraser (Takara, Cat#RR037A) to eliminate genomic DNA contamination.

Quantitative PCR was conducted on a StepOnePlus™ Real-Time PCR System (Applied Biosystems) using TB Green^®^ Premix Ex Taq™ II (Takara, Cat#RR820A). Each 20 μL reaction contained 10 μL of PCR master mix, 0.4 μL of each primer (10 μM)) (primer detailed in **Supplementary Table 1**), 2 μL of cDNA, and nuclease-free water. The thermal cycling conditions consisted of an initial denaturation at 95°C for 30 seconds, followed by 40 amplification cycles at 95°C for 5 seconds and 60°C for 30 seconds. Specificity of amplification was confirmed via melt curve analysis.

Gene expression was normalized to GAPDH using the 2^−^ΔΔCt method, and all reactions were performed in technical triplicates.

### 2.11 Cell proliferation and colony formation assays

Cell proliferation was assessed using the Cell Counting Kit-8 (CCK-8; Dojindo, Cat# CK04-500) following the manufacturer’s instructions. Briefly, 5 × 10³ cells per well were seeded into 96-well plates, and cell viability was evaluated at 0, 24, 48, 72, and 96 hours post-seeding. At each time point, 10 µL of CCK-8 reagent was added to each well and incubated for 2 hours at 37°C. Absorbance was recorded at 450 nm using a BioTek microplate reader. All experimental conditions were analyzed in triplicate.

For clonogenic assays, 500 cells per well were plated into 6-well plates and maintained under standard culture conditions for 10–14 days to allow colony formation. Colonies were then fixed with 100% methanol for 20 minutes, stained with 0.5% crystal violet solution (Beyotime, Cat# C0121) for 15 minutes, and rinsed with water. Colonies containing more than 50 cells were counted manually under an inverted microscope. All experiments were independently repeated in triplicate.

### 2.12 Wound healing and transwell migration assays

Cell migratory capacity was evaluated through wound healing and Transwell migration assays. For the scratch assay, cells were seeded into 6-well plates and cultured until reaching approximately 90% confluence. A linear wound was generated across the monolayer using a sterile 200-μL pipette tip, followed by gentle rinsing with phosphate-buffered saline (PBS) to remove cellular debris. Cells were then incubated in serum-free medium, and wound closure was documented at 0 and 72 hours using an inverted phase-contrast microscope (Olympus IX73). The wound area was quantified using ImageJ software.

For Transwell migration assays, 1 × 10^5^ cells suspended in serum-free medium were placed into the upper chamber of a Transwell insert with an 8-μm pore membrane (Corning, Cat# 3422). The lower chamber was filled with medium containing 10% fetal bovine serum (FBS) to serve as a chemoattractant. After 12 hours of incubation at 37°C, non-migratory cells were gently removed from the upper surface of the membrane. The inserts were then fixed with 4% paraformaldehyde (Abcam, Cat# ab185002) and stained using 0.5% crystal violet (Cell Signaling Technology, Cat# 14631). Migrated cells adherent to the underside of the membrane were imaged and counted in five randomly selected microscopic fields.

### 2.13 ELISA for cytokine secretion

To evaluate cytokine secretion, cells were exposed to 1 µg/mL lipopolysaccharide (LPS; Abcam, Cat# ab203508) for 24 hours. Following stimulation, cell culture supernatants were collected and clarified by centrifugation to remove residual debris. The concentrations of tumor necrosis factor-alpha (TNF-α) and interleukin-6 (IL-6) were measured using commercially available ELISA kits (TNF-α: Abcam, Cat# ab100747; IL-6: Abcam, Cat# ab178013), in accordance with the manufacturer’s protocols. Optical density was measured at 450 nm using a microplate spectrophotometer. All experimental conditions were assessed in triplicate to ensure data reproducibility.

### 2.14 Statistical analysis

All statistical analyses were conducted using R software (version 4.1.3) for omics-related datasets and GraphPad Prism (version 8.0) for experimental data analysis and graphical visualization. Data are expressed as mean ± standard deviation (SD), with each experiment independently repeated at least three times to ensure reproducibility. Comparisons between two groups were performed using unpaired two-tailed Student’s *t*-tests, while multiple group comparisons were evaluated via one-way ANOVA followed by Tukey’s *post hoc* test. Statistical significance was defined as *P* < 0.05, with the following annotations: *P < 0.05; **P < 0.01; ***P < 0.001.

## 3 Results

### 3.1 Single-cell transcriptomic landscape of traumatic brain injury in mice

**Figure 1:**
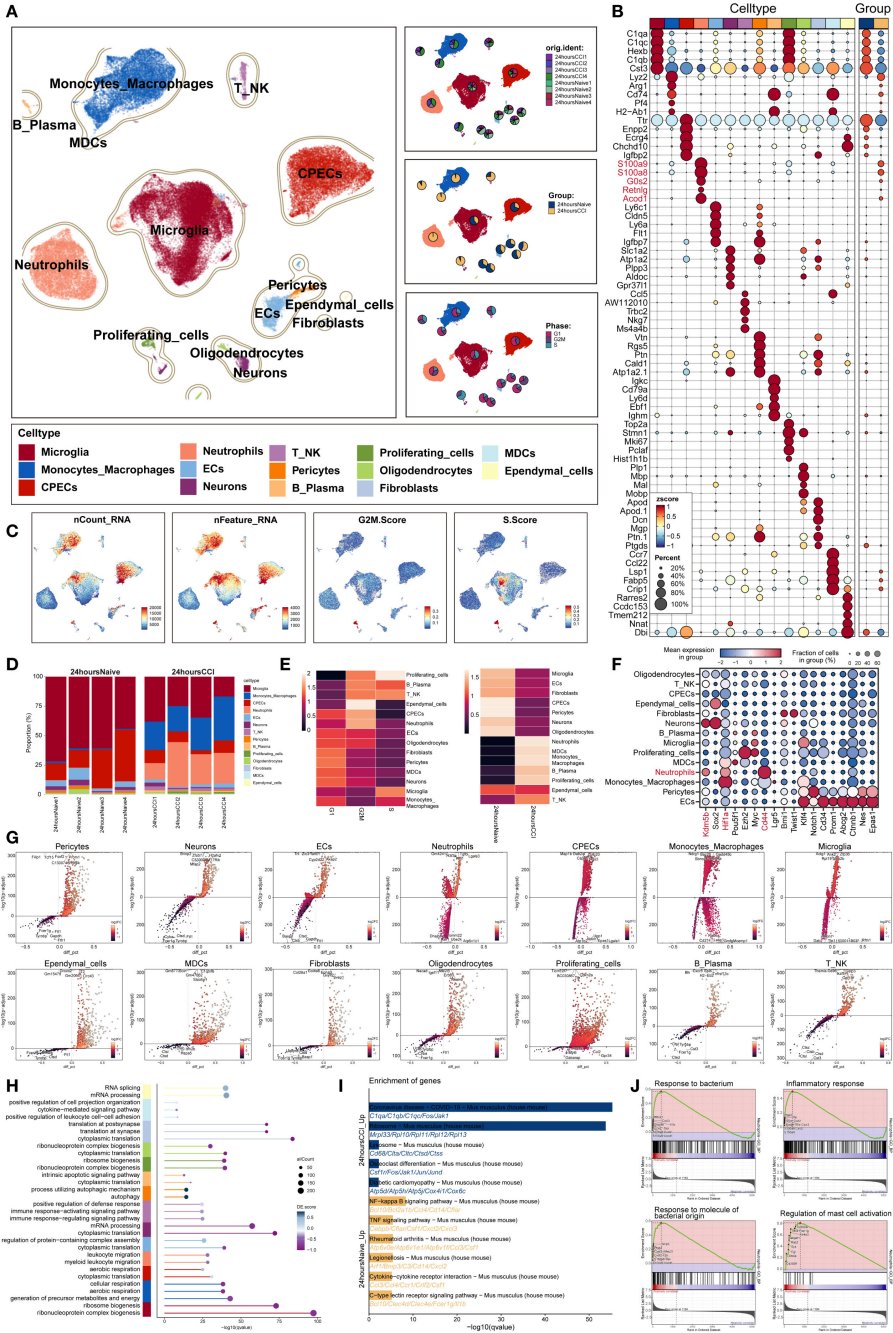
Single-cell transcriptomic panorama analysis of the heterogeneity in the TBI microenvironment. **(A)** UMAP visualization of the distribution characteristics of all cell samples based on specific marker genes for cell type identification and annotation. The three-panel figure on the right shows the proportions of the sample group, tissue origin group (24 hours native and 24 hours CCI), and cell cycle (G1, G2/M, S phase) in each cell type. **(B)** Bubble plot analyzed the average expression levels of the top five marker genes in each cell type and tissue origin, with bubble size proportional to gene expression percentage, and the color gradient indicating data normalization. **(C)** Distribution characteristics of nCount RNA, nFeature RNA, G2/M.Score, and S.Score. **(D)** Stacked bar chart quantified the differences in cell composition between the 24 hours native and 24 hours CCI groups. **(E)** Ro/e scores revealed the preference of cell types for different cell cycles (left) and cell origin groups (right). **(F)** Bubble plot revealed the differential expression of stemness feature genes across different cell types. **(G)** Volcano plot visualized DEGs in various TBI cell types, highlighting the top five upregulated and downregulated genes in each cell type (P-adj < 0.05), with dashed lines marking the significance boundary. **(H)** GO enrichment analysis of different biological processes for each cell type. **(I)** KEGG enrichment analysis of differential genes based on each cell type between 24 hours native and 24 hours CCI groups. **(J)** GSEA enrichment analysis of neutrophils.

To delineate the cellular architecture of TBI, we analyzed single-cell RNA sequencing data (GSE269748) derived from eight murine brain samples—comprising four sham-operated and four controlled cortical impact (CCI) models harvested 24 hours post-injury. Following rigorous quality control and unsupervised clustering, a total of 67,993 high-quality cells were retained for downstream characterization (**Figure 1A**).

Using established marker genes, we annotated 14 transcriptionally distinct cell populations, broadly categorized into: (1) central nervous system (CNS)–resident cells (including microglia, choroid plexus epithelial cells (CPECs), neurons, oligodendrocytes, and ependymal cells); (2) vascular-associated components (endothelial cells, pericytes, and fibroblasts); and (3) immune infiltrates (comprising monocytes/macrophages, neutrophils, T/NK cells, B/plasma cells, myeloid dendritic cells [MDCs], and proliferating cells). Notably, the frequency of neutrophils and monocytes was markedly increased in the TBI group, indicating robust early immune mobilization.

Cell cycle analysis revealed that neutrophils predominantly occupied the G2/M phase, whereas microglia were enriched in the S phase. Differential gene expression profiling demonstrated marked upregulation of neutrophil-related transcripts, such as S100a8, S100a9, G0s2, Retnlg and Acod1, in TBI-affected brains (**Figure 1B**), consistent with their activation and expansion.

Increased transcriptional activity was also evident in microglia, CPECs, monocytes/macrophages, and proliferative clusters, reflected by elevated nCount-RNA and nFeature-RNA values. Neutrophils displayed strong transcriptional signatures, reinforcing their early functional involvement (**Figure 1C**). Quantitative evaluation confirmed a significant rise in neutrophil abundance in TBI samples (**Figure 1D**).

Furthermore, observed-to-expected ratio (Ro/e) analysis demonstrated selective neutrophil enrichment and preferential G2/M phase accumulation post-injury (**Figure 1E**), highlighting their pivotal role in shaping the acute neuroinflammatory microenvironment following traumatic insult.

### 3.2 Stemness signatures and functional enrichment of cell populations following TBI revealed by single-cell transcriptomics

To evaluate stem-like properties among diverse brain cell populations post-trauma, we profiled the expression of genes associated with cellular stemness across annotated clusters using bubble plot visualization. Neutrophils demonstrated elevated expression of canonical stemness regulators, including Kdm5b, Hif1a, and CD44, suggesting the potential acquisition of progenitor-like traits within this population (**Figure 1F**).

A global transcriptomic comparison using volcano plots highlighted the top five significantly upregulated and downregulated genes in each of the 14 identified cell types (**Figure 1G**). In neutrophils, genes such as Gm42418, H3f3b, Pfn1, Tgfbi, and Lgals3 were among the most upregulated and likely implicated in cellular activation and tissue remodeling. In contrast, decreased expression of Dnajb6, Rer1, Tomm22, Ube2k, and Atp6v1c1 may reflect context-dependent suppression or metabolic reprogramming in response to the post-injury milieu.

To elucidate the biological roles of neutrophil-enriched differentially expressed genes (DEGs), we performed Gene Ontology (GO) analysis, which revealed significant enrichment in inflammation-associated processes such as leukocyte chemotaxis and myeloid cell migration (**Figure 1H**), underscoring their mobilization and recruitment to sites of injury.

Kyoto Encyclopedia of Genes and Genomes (KEGG) pathway analysis indicated activation of key proinflammatory cascades, particularly the NF-κB and TNF signaling pathways (**Figure 1I**), both of which are critical for acute innate immune responses.

In addition, gene set enrichment analysis (GSEA) revealed that neutrophils were significantly enriched in immune-related pathways (**Figure 1J**), including defense against bacterial challenge, generalized inflammatory signaling, and mast cell–mediated processes.

### 3.3 Heterogeneity of neutrophil subsets following TBI and molecular characterization

**Figure 2:**
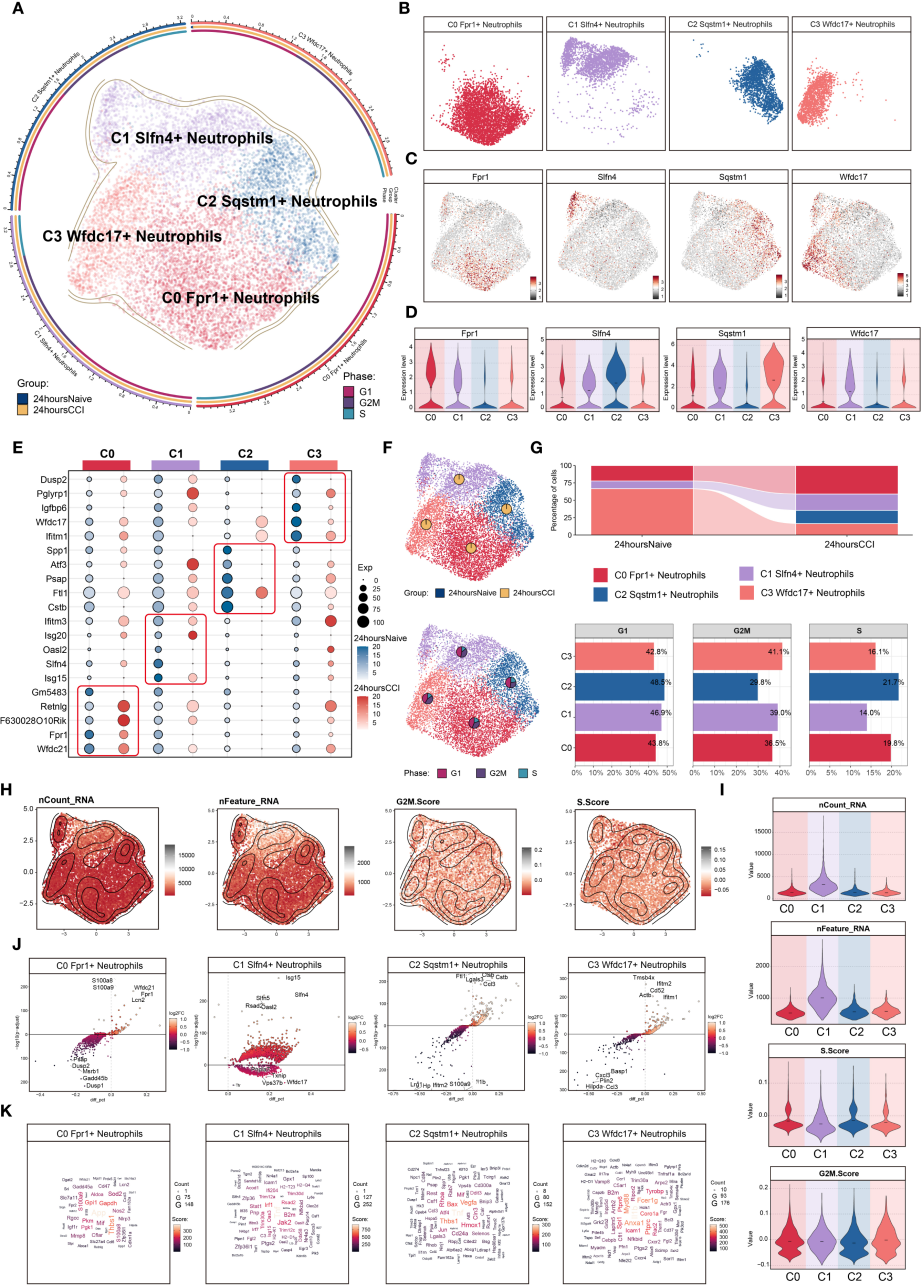
Characterization of neutrophil subpopulation profiles after TBI. **(A)** UMAP plot presented the clustering distribution characteristics of four distinct neutrophil subpopulations identified based on differential marker gene expression, with contour lines outlining the boundaries of each subpopulation. The outer axis represented the log scale of each neutrophil subpopulation. The three-ring annotation layer encoded subpopulation classification (outer ring), tissue origin group (middle ring: 24 hours native vs. 24 hours CCI), and cell cycle stage (inner ring: G1, G2/M, S phase). **(B)** Faceted UMAP plot compared the distribution characteristics of individual neutrophil subpopulations. **(C, D)** UMAP visualization of the distribution patterns of specific marker genes for each neutrophil subpopulation, with violin plots showed their expression levels across subpopulations. **(E)** Bubble plot analyzed the average expression levels of the top five marker genes in each neutrophil subpopulation, with bubble size proportional to gene expression percentage, and the color gradient indicating data normalization. **(F)** UMAP plot combined with pie charts to display the distribution proportions of the 24 hours native and 24 hours CCI groups (top) and cell cycle phases (G1, G2/M, S phase, bottom) across the four neutrophil subpopulations. **(G)** Proportional chart quantified the composition of each neutrophil subpopulation in different cell origin groups (top) and cell cycle phases (bottom). **(H, I)** UMAP plot combined with violin plots showed the distribution characteristics and expression levels of nCount RNA, nFeature RNA, G2/M.Score, and S.Score. **(J)** Volcano plot visualized DEGs in neutrophil subpopulations, highlighting the top five upregulated and downregulated genes in each subpopulation (P-adj < 0.05), with dashed lines marking the significance boundary. **(K)** Word cloud constructed based on GO BP enrichment analysis, visually representing the enrichment degree of genes associated with neutrophil subpopulations.

To dissect the heterogeneity of neutrophils involved in the acute phase of TBI, we focused on 9,553 neutrophils isolated at 24 hours post-injury. Unsupervised clustering delineated four transcriptionally distinct neutrophil subsets (**Figure 2A**): C0 (Fpr1 high), C1 (Slfn4 high), C2 (Sqstm1 high), and C3 (Wfdc17 high). Spatial visualization using UMAP facet plots revealed that each subset exhibited unique localization patterns within the embedding space (**Figure 2B**), suggesting divergent functional identities. Representative expression profiles of signature genes are shown in **Figures 2C, D**.

A bubble plot summarizing the top five marker genes for each cluster confirmed distinctive transcriptional signatures: C0 (Fpr1^+^): Wfdc21, Fpr1, F630028O10Rik, Retnlg, and Gm5483; C1 (Slfn4^+^): Isg15, Slfn4, Oasl2, Isg20, and Ifitm3; C2 (Sqstm1^+^): Cstb, Ftl1, Psap, Atf3, and Spp1; C3 (Wfdc17^+^): Ifitm2, Wfdc17, Igfbp6, Pglyrp1, and Dusp2 (**Figure 2E**).

Analysis of sample origin (**Figure 2F**) demonstrated that nearly all neutrophils were derived from the injured (CCI) group, with negligible representation in the sham control brains. Cell cycle analysis revealed no significant differences in G1, S, or G2/M phase distributions among the subsets, which is consistent with the generally non-proliferative nature of mature neutrophils in brain parenchyma.

Proportional distribution analysis indicated a marked expansion of the C0 (Fpr1^+^), C1 (Slfn4^+^), and C2 (Sqstm1^+^) subsets following TBI, whereas the C3 (Wfdc17^+^) subset was notably diminished (**Figure 2G**). Interestingly, the C1 (Slfn4^+^) population exhibited the highest transcriptional activity, as evidenced by elevated nCount-RNA and nFeature-RNA values (**Figures 2H, I**), although no significant enrichment was observed in proliferative cell cycle phases.

Differential gene expression analysis using volcano plots revealed that the C1 cluster was specifically enriched for inflammatory and interferon-responsive transcripts, including Isg15, Slfn4, Slfn5, Rsad2, and Oasl2, while genes such as Pnpla7, Txnip, Vps37b, Wfdc17, and Ttr were downregulated (**Figure 2J**). A DEG word cloud visualization emphasized key immune mediators such as Tnf and Irf1, suggesting that the C1 (Slfn4^+^) neutrophils may represent a transcriptionally activated subpopulation with immunoregulatory functions in the early TBI microenvironment (**Figure 2K**).

### 3.4 N1-like polarization and metabolic plasticity of SLFN4^+^ neutrophils post-TBI

To delineate the immunophenotypic polarization and metabolic adaptations of the SLFN4^+^ neutrophil subset in the context of TBI, we performed comprehensive gene set activity analyses. Considering the dynamic capacity of neutrophils to adopt either pro-inflammatory (N1) or anti-inflammatory (N2) phenotypes in neuroinflammatory environments, we utilized the AUCell scoring algorithm to quantify the expression of polarization-associated gene modules.

**Figure 3:**
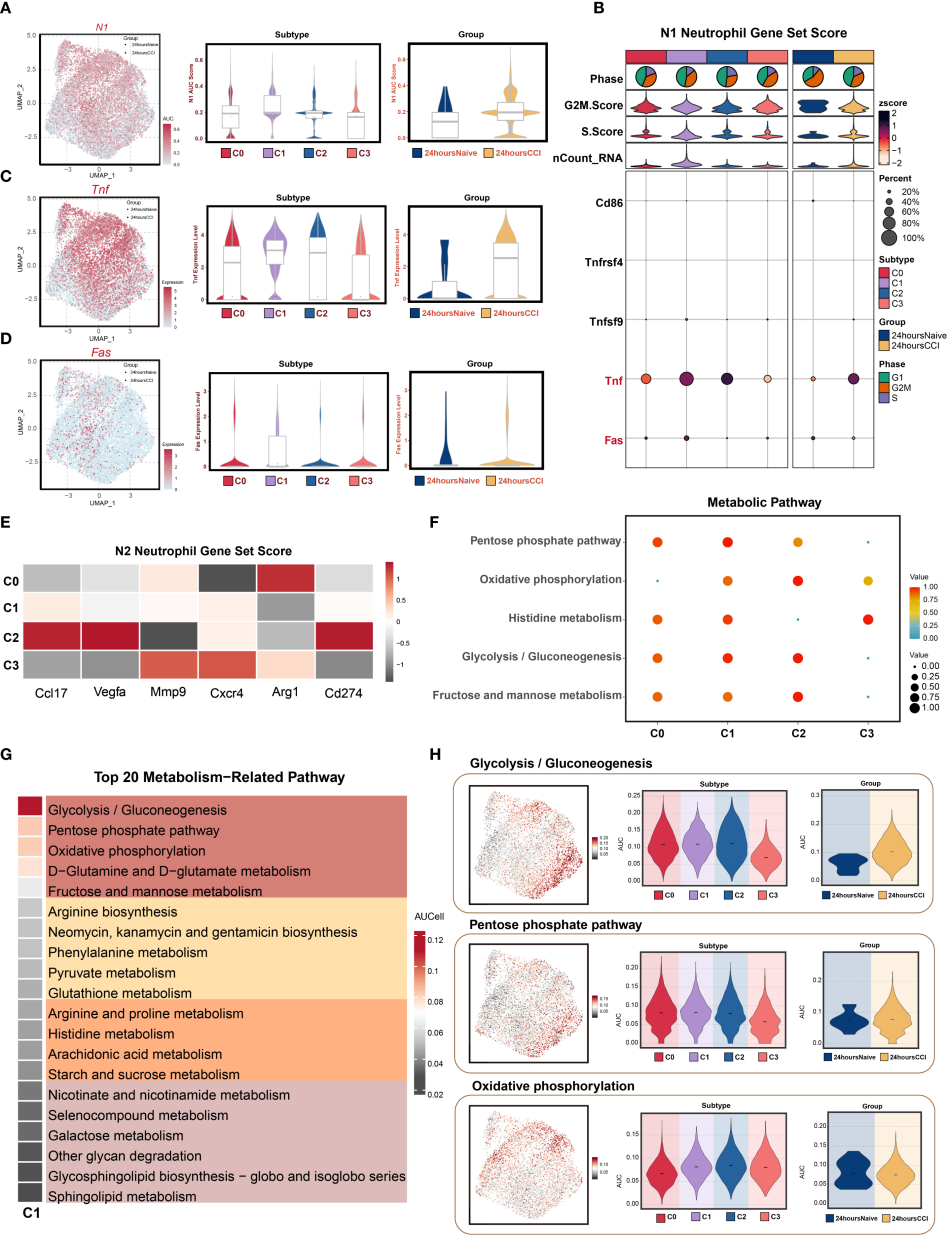
N1 polarization features and metabolic reprogramming of Slfn4*+* neutrophil subpopulation. **(A)** AUCell algorithm was used to calculate the activity levels of the N1 neutrophil feature gene set in each cell subpopulation, visualized using UMAP. Geometric markers differentiate tissue origins, with circles representing the 24 hours native group and triangles representing the 24 hours CCI group. The violin plot on the right quantitatively characterized the differential expression features of the N1 neutrophil gene set activity score across subpopulations and tissue origins, showing median and quartile range statistics. **(B)** Analysis of the expression differences of N1 neutrophil-related genes based on average gene expression levels across subpopulations and tissue origins. **(C, D)** UMAP analysis revealed the distribution patterns of significantly highly expressed N1 neutrophil feature genes (e.g., Tnf, Fas) in the C1 Slfn4+ neutrophil subpopulation. Violin-box plots further analyze the expression level heterogeneity of these genes across different neutrophil subpopulations and tissue origins. **(E)** Analysis of the expression differences of N2 neutrophil-related genes based on average gene expression levels across subpopulations and tissue origins. **(F)** Bubble plot visually represented the significantly enriched metabolic pathways across neutrophil subpopulations. **(G)** Heatmap showed the top 20 metabolic pathways in the C1 Slfn4+ neutrophil subpopulation. **(H)** UMAP analysis revealed the distribution patterns of significantly highly expressed metabolic pathways (Glycolysis/Gluconeogenesis, Pentose phosphate pathway, Oxidative phosphorylation) in the C1 Slfn4+ neutrophil subpopulation. Violin plots further analyze the expression level heterogeneity of these pathways across different neutrophil subpopulations and tissue origins.

As depicted in **Figures 3A–D**, SLFN4^+^ neutrophils demonstrated robust enrichment of N1-associated gene signatures, including markedly upregulated expression of classical pro-inflammatory markers such as TNF and FAS. In particular, Tnf expression was significantly higher in the CCI group compared to the sham controls, indicating a heightened inflammatory status in these cells. Conversely, N2-associated transcripts such as CXCR4, ARG1, and CD274 were comparatively diminished in this population (**Figures 3A, E**), suggesting a skewing toward an N1-like activation profile. These findings position the C1 (SLFN4^+^) subpopulation as a key contributor to the early pro-inflammatory milieu following traumatic injury to the brain.

To explore the metabolic underpinnings of this inflammatory phenotype, we conducted pathway enrichment analyses of metabolic gene sets. The C1 cluster displayed significant enrichment in energy metabolism–related pathways, including glycolysis/gluconeogenesis, pentose phosphate pathway, oxidative phosphorylation, histidine metabolism, and fructose and mannose metabolism (**Figure 3F**). Among the top 20 enriched metabolic pathways, glycolytic and pentose phosphate pathways, along with D-glutamine and D-glutamate metabolism, emerged as dominant (**Figure 3G**), suggesting metabolic reprogramming aligned with heightened inflammatory activity.

UMAP projections illustrated the spatial distribution of metabolic pathway activity within the neutrophil compartment, while violin plots confirmed that glycolysis/gluconeogenesis and pentose phosphate pathway activity were significantly upregulated in the CCI group at 24 hours post-injury (**Figure 3H**). These data imply that bioenergetic reprogramming, particularly increased glycolytic flux and oxidative metabolism, may support the effector functions of SLFN4^+^ neutrophils under acute neuroinflammatory conditions.

### 3.5 Stemness-driven trajectories reveal dynamic neutrophil state transitions in early TBI

**Figure 4:**
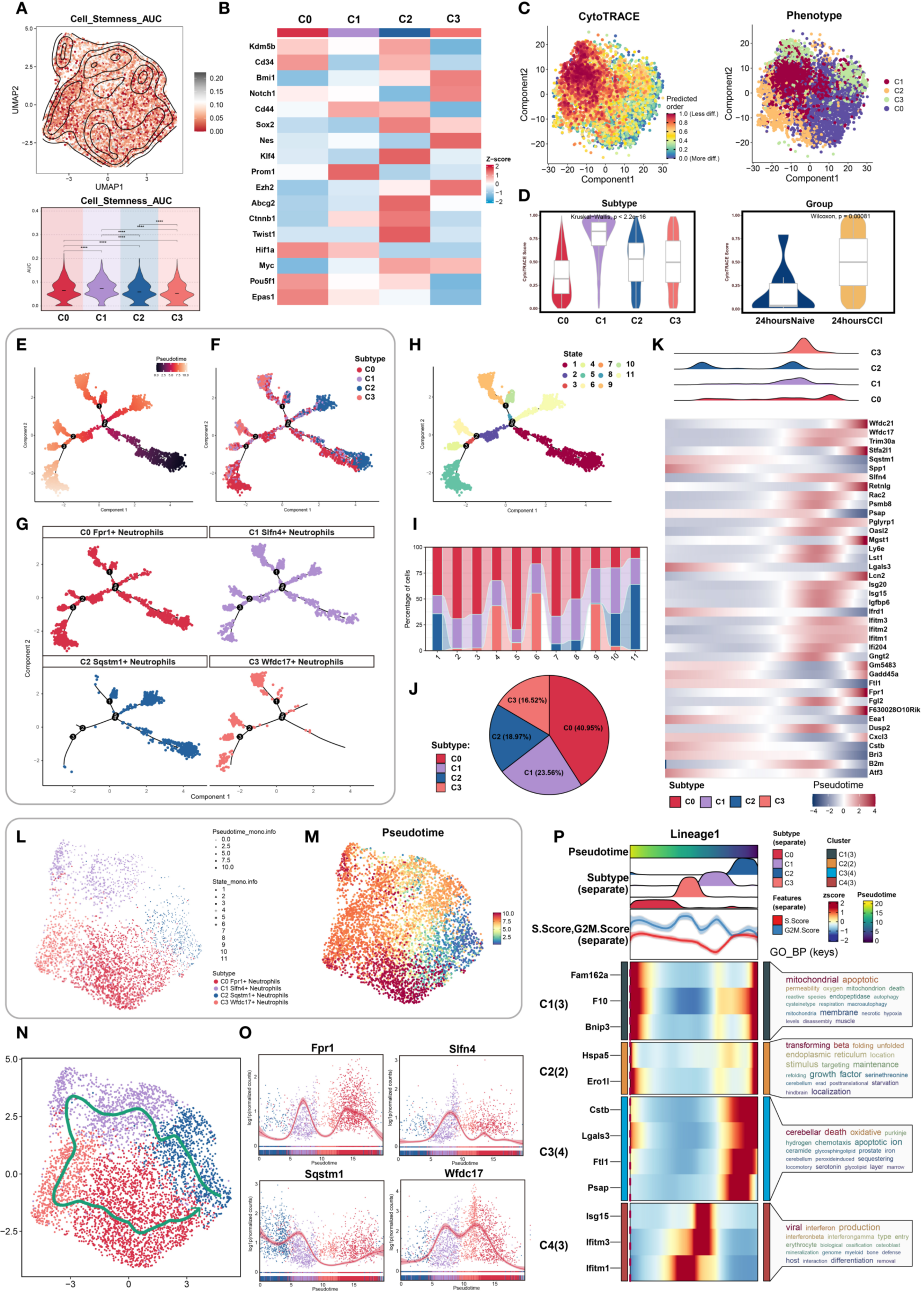
Stemness heterogeneity and pseudotime dynamics analysis of neutrophil subpopulations. **(A)** UMAP combined with contour plots showcased the distribution differences in stemness AUC values, with a violin plot quantifying the heterogeneity among neutrophil subpopulations. **(B)** Differential expression of stemness genes across neutrophil subpopulations. **(C)** (Left) Cell stemness prediction distribution based on the CytoTRACE algorithm, with a color gradient reflecting stemness index (0-1, red: high stemness, blue: low stemness); (Right) UMAP plot showed neutrophil subpopulation distribution, color-coded for different neutrophil subpopulations. **(D)** CytoTRACE analysis quantified stemness across different neutrophil subpopulations and tissue origins. **(E–G)** Monocle analysis inferred the developmental trajectory of neutrophils, visualized by pseudotime with facet plots showing the positioning of different cell types along the developmental trajectory. **(H, I)** Ordering pseudotime states, considering the proportions of neutrophil subpopulations at each of the nine time points. **(J)** Pie chart summarized the proportions of each neutrophil subpopulation. **(K)** Heatmap showed the differential gene expression of neutrophil subpopulations as pseudotime progresses. **(L, M)** UMAP plot showed the dynamic changes in the pseudotime trajectory of neutrophil subpopulations, with point shapes indicating different state classifications. **(N)** Slingshot algorithm was used to construct the pseudotime trajectory, visualized by UMAP to show the differentiation path of neutrophil subpopulations, with arrows indicating the direction of differentiation progression. **(O)** Expression levels of four marker genes in neutrophil subpopulations showed temporal changes. **(P)** Dynamic expression profiles of marker genes compared the temporal trends of different genes.

To dissect the intra-lineage plasticity and transitional dynamics of neutrophils during early TBI, we began by evaluating stemness potential across identified subclusters. Using AUCell-based scoring, the C1 neutrophil subset exhibited the highest stemness activity among all groups (**Figure 4A**), indicating a pronounced regenerative or progenitor-like profile. This finding suggested that C1 neutrophils may represent an early-stage regulatory population involved in the acute immune response post-injury.

We then examined the distribution of canonical stemness-associated genes across clusters. Heatmap analysis revealed preferential enrichment of Prom1, a well-established stemness marker, in the C1 subpopulation, further supporting its potential progenitor role (**Figure 4B**).

To map the temporal progression of neutrophil states, we performed pseudotime analysis. CytoTRACE was used to establish an initial developmental hierarchy based on transcriptional diversity, revealing that cells from the C1 cluster and the CCI group at 24 hours post-injury were enriched at the early pseudotime axis, corresponding to higher inferred stemness (**Figures 4C, D**). These data suggested that C1 cells may serve as intermediates in the early neutrophil response to TBI.

Using Monocle, we constructed high-resolution pseudotemporal trajectories that delineated 11 transcriptional states spanning early progenitor-like to terminally differentiated cells. These states segregated into three distinct branches (**Figures 4E, H**). Subpopulation mapping across the pseudotime continuum showed that C2 neutrophils were positioned predominantly at the origin of the trajectory, whereas C1 and C3 cells occupied intermediate zones, and C0 neutrophils were enriched at terminal states (**Figures 4F, G, I**). The relative distribution of these subsets was visualized by pie chart, indicating C0 as the most prevalent, followed by C1 (**Figure 4J**).

We then analyzed the temporal expression of differentially expressed genes along the trajectory. Heatmaps and trend plots highlighted sequential activation and repression programs corresponding to specific developmental transitions (**Figures 4K–M**), reflecting dynamic transcriptional remodeling during neutrophil maturation in response to injury.

To corroborate trajectory structure, we implemented Slingshot analysis, which identified a primary lineage extending from C2 to C0, consistent with Monocle findings (**Figure 4N**). Expression trend visualization along this lineage further affirmed the temporal gene expression patterns (**Figure 4O**).

Finally, Gene Ontology enrichment of trajectory-resolved gene modules revealed stage-specific biological processes enriched at distinct phases of differentiation. These included inflammatory activation, migration, and metabolic adaptation pathways, providing mechanistic insights into how neutrophil subpopulations contribute functionally to TBI pathology (**Figure 4P**).

### 3.6 Functional heterogeneity of neutrophil subsets uncovered by GO and pathway enrichment

**Figure 5:**
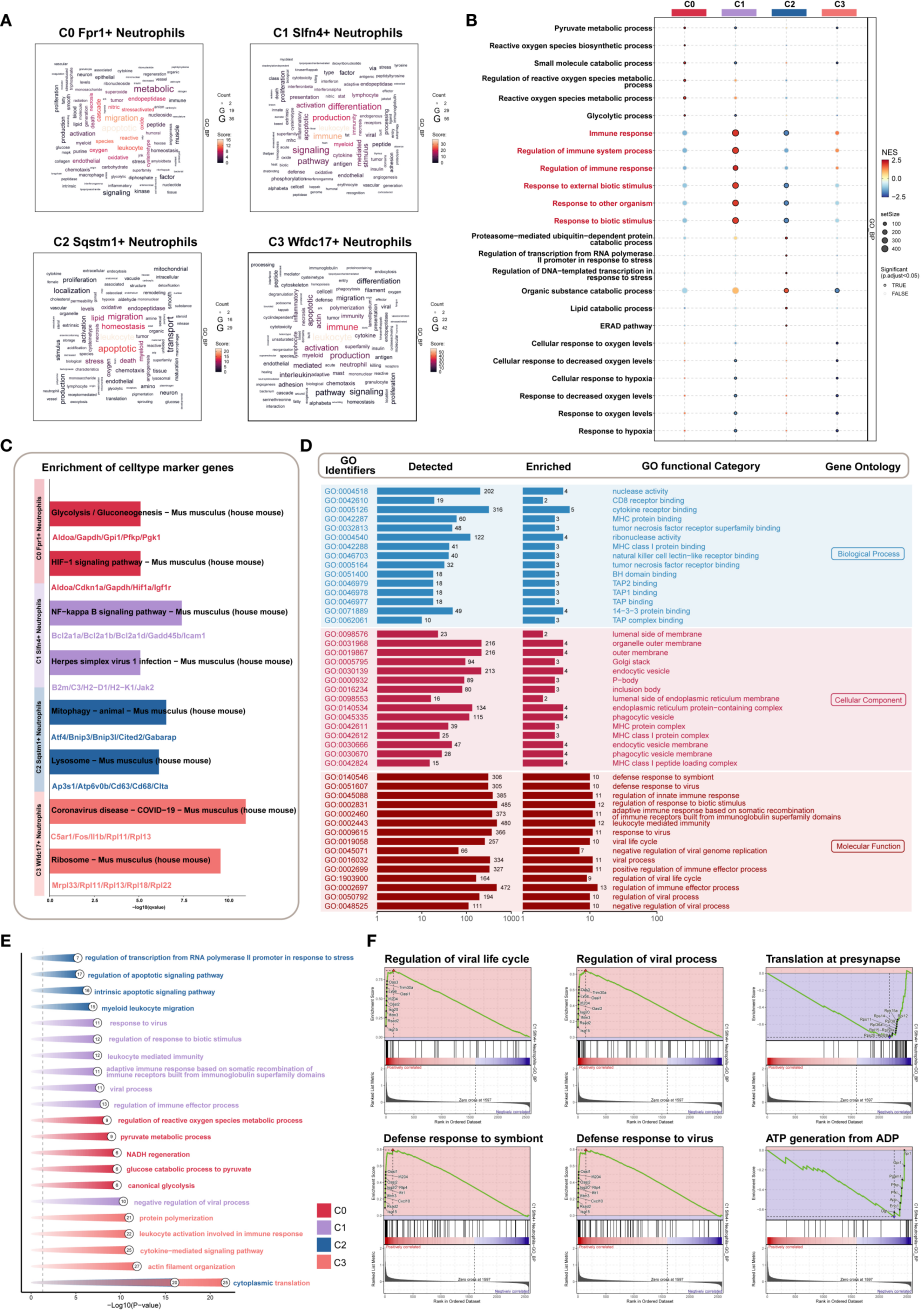
Biological features of *Slfn4+* subpopulation based on GO/KEGG/GSEA enrichment strategies. **(A)** Word cloud constructed from GOBP enrichment analysis, visually showing the enrichment degree of pathways associated with neutrophil subpopulations. **(B)** Bubble plot showed the results of GSEA enrichment analysis of gene sets in different neutrophil subpopulations, revealing key biological functions in each subpopulation. **(C)** KEGG pathway enrichment based on the marker genes of each neutrophil subpopulation. **(D)** GOBP, GOCC, GOMF enrichment analysis of Slfn4+ neutrophil subpopulation. **(E)** GO analysis based on differential genes of the four neutrophil subpopulations. **(F)** GSEA enrichment analysis of the Slfn4+ neutrophil subpopulation.

To elucidate the distinct functional profiles of neutrophil subpopulations in the acute phase of TBI, we performed comprehensive Gene Ontology (GO) and Kyoto Encyclopedia of Genes and Genomes (KEGG) enrichment analyses. GO Biological Process (GOBP) terms revealed divergent functional signatures among the four clusters (**Figure 5A**). The C0 cluster was predominantly associated with apoptotic regulation and cell migration. In contrast, C1 neutrophils were enriched in leukocyte activation and immune effector responses, implying a pivotal immunoregulatory role. C2 cells showed enrichment in pathways related to innate immunity and tissue homeostasis, whereas C3 shared immunological characteristics with C1 but with reduced activation signatures.

To further distinguish C1 from C3, Gene Set Enrichment Analysis (GSEA) was conducted. C1 neutrophils demonstrated specific upregulation of immune-related gene sets such as “immune response” and “response to external stimulus” (**Figure 5B**), underscoring their heightened involvement in the early host defense mechanisms following TBI.

KEGG pathway analysis highlighted significant enrichment of C1 cells in pro-inflammatory signaling cascades, including the NF-κB signaling axis and antiviral defense pathways such as “Herpes simplex virus 1 infection” (**Figure 5C**). These findings suggest that the C1 subpopulation plays a key role as an inflammatory amplifier within the post-traumatic immune landscape.

Additional GO enrichment across all three ontology domains—biological process (BP), cellular component (CC), and molecular function (MF)—further characterized C1 cells. Enriched terms included cytokine receptor binding and nuclease activity (BP), localization to outer membranes and secretory vesicles (CC), and immunomodulatory molecular functions (MF) (**Figure 5D**). Differential gene expression-based enrichment corroborated these results, further linking C1 cells to key immune defense mechanisms (**Figure 5E**).

Moreover, GSEA revealed positive enrichment of antiviral responses, including “defense response to virus” and “regulation of viral life cycle,” within C1 neutrophils. Conversely, metabolic pathways, particularly those involved in ATP synthesis and protein translation at synaptic terminals, were negatively enriched (**Figure 5F**). This suggests a functional trade-off, whereby C1 neutrophils prioritize immune activation over metabolic maintenance in the context of acute inflammation.

Taken together, these analyses position the C1 (SLFN4^+^) neutrophil subset as a functionally specialized and immunologically dominant population during the early stages of TBI. Its dual engagement in antiviral and pro-inflammatory pathways highlights its potential as a therapeutic target for modulating neuroinflammation while preserving essential innate immune functions.

### 3.7 Intercellular communication highlights the central signaling role of the SLFN4^+^ neutrophil subset in early post-TBI inflammation

**Figure 6:**
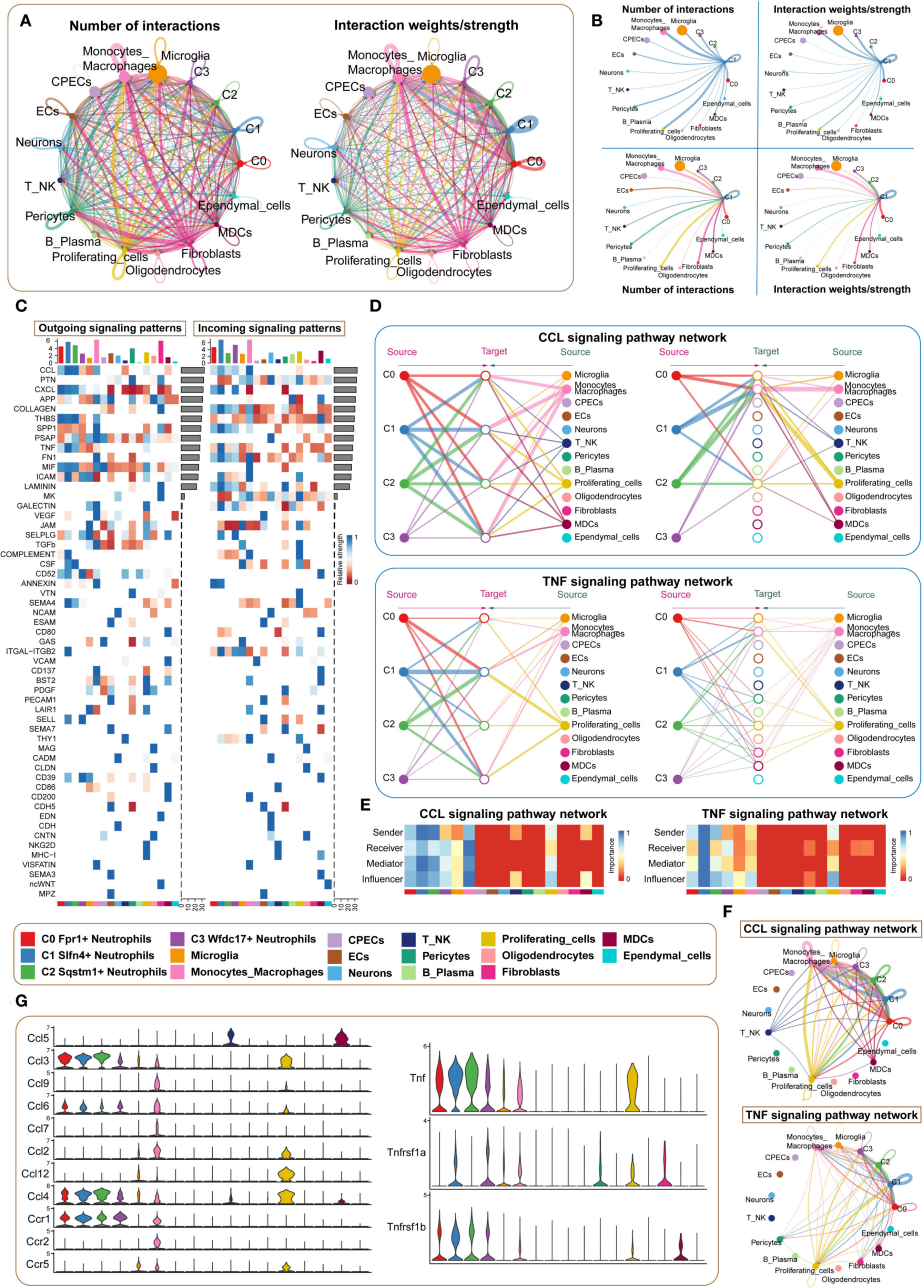
Microenvironment interaction network of *Slfn4+* neutrophil subpopulation mediating CCL/TNF signaling pathways. **(A)** Circos plots showed the number (upper) and strength (lower) of interactions between neutrophil subpopulations and microenvironmental cells, with point size representing interaction quantity and line thickness reflecting communication probability. **(B)** Interaction analysis of Slfn4+ neutrophils with other cells: the top left and top right circle plots showed interaction numbers and weights when acting as signal senders; the bottom left and bottom right circle plots showed interaction numbers and weights when acting as signal receivers. **(C)** Heatmap of outgoing and incoming signaling patterns for neutrophil subpopulations. **(D)** Hierarchical diagrams showed interactions between neutrophil subpopulations and other cell types in the CCL and TNF signaling pathways. **(E)** Heatmap showed the cell communication centrality scores for CCL and TNF signaling pathways. **(F)** CCL and TNF signaling communication network circle plot. **(G)** Violin plot compared the ligand-receptor protein activity differences in the CCL and TNF pathways between neutrophil subpopulations and other cell types.

To further investigate the intercellular dynamics contributing to neuroinflammation following traumatic brain injury, we performed a comprehensive cell–cell communication analysis centered on the SLFN4^+^ (C1) neutrophil subpopulation. While prior transcriptomic profiling underscored the immunological relevance of this cluster, its functional interactions with other immune and stromal compartments required further delineation. Using ligand–receptor inference modeling, we mapped a global communication network across 17 cell types, including four neutrophil subsets and 13 additional populations. Chord diagrams illustrated the overall communication topology and interaction intensity within the injured brain microenvironment (**Figures 6A, B**). Notably, the C1 cluster demonstrated strong bidirectional signaling with multiple immune subsets, particularly monocytes and macrophages. Heatmap analyses of outgoing and incoming signaling pathways identified the CCL and TNF axes as dominant mediators of intercellular exchange (**Figure 6C**). Dissecting these pathways further, we observed that in the CCL network, C1 neutrophils primarily served as signal initiators and modulators, while monocyte-derived cells were the principal recipients (**Figures 6D, E**). Interestingly, within the TNF signaling cascade, C1 cells exhibited a multifaceted role, functioning concurrently as signal senders, receivers, and mediators, indicating their integrative capacity in amplifying local inflammatory responses. Network topology visualizations confirmed that C1 neutrophils acted as key nodes in propagating paracrine and autocrine cues (**Figure 6F**), while violin plots of core ligand–receptor pairs highlighted the selective expression of interaction mediators such as Ccl3–Ccr1 and Tnf–Tnfrsf1b across cell types (**Figure 6G**). Collectively, these findings position the SLFN4^+^ neutrophil subpopulation as a central orchestrator of inflammatory signaling in early TBI, exerting regulatory influence over surrounding immune cells through sustained activation of chemokine and cytokine pathways. These insights may offer a mechanistic framework for targeting neutrophil-derived signaling in the therapeutic modulation of post-traumatic neuroinflammation.

### 3.8 Transcriptional regulatory landscape of neutrophil subpopulations revealed by PySCENIC analysis

**Figure 7:**
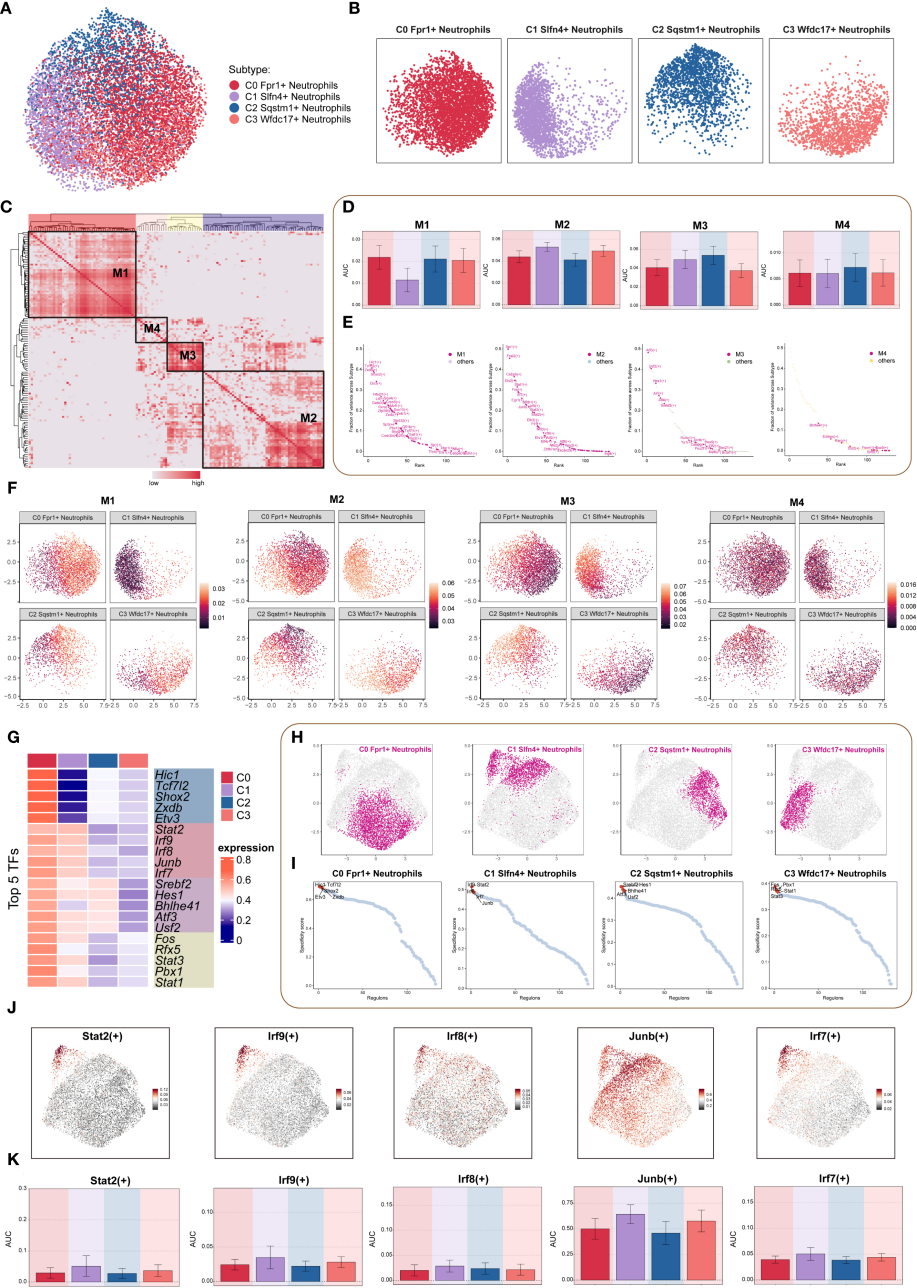
TF regulatory networks in neutrophil subpopulations. **(A, B)** Dimensionality reduction clustering of neutrophil subpopulations based on all TF regulatory activities, visualized by faceted UMAP plots to show the distribution features of each subpopulation. **(C)** Heatmap based on SCENIC-identified transcription module similarity, using AUCell scores to identify four regulon modules (M1-M4) in neutrophil subpopulations. **(D)** Bar chart quantified the AUC score differences across neutrophil subpopulations for each of the four modules. **(E)** Scatter plot ranking TFs based on variance scores in each regulatory module, highlighting key regulatory factors. **(F)** Faceted UMAP plot showed the distribution features of four regulatory modules across different neutrophil subpopulations. **(G)** Heatmap showed the top 5 TFs in each neutrophil subpopulation. **(H)** Faceted UMAP plots presented the distribution features of each neutrophil subpopulation. **(I)** The scatter plot ranked the TFs of each neutrophil subpopulation according to their Regulon specificity score, highlighting the top 5 ranked TFs. **(J, K)** The UMAP plot visualized key TFs (Stat2(+), Irf9(+), Irf8(+), Junb(+), Irf7(+)) in the Slfn4+ neutrophil subpopulation, and bar plots compared the activity differences of these TFs between subpopulations.

To dissect transcriptional regulatory heterogeneity among neutrophil subtypes following TBI, we applied the pySCENIC pipeline to construct transcription factor (TF)-centered gene regulatory networks at single-cell resolution. This analysis enabled the identification of regulon activity patterns, which were used to stratify cells based on transcriptional control mechanisms. Clustering based on both transcriptomic profiles (**Figures 7A, B**) and regulon activation scores (**Figure 7C**) delineated four distinct regulatory modules. Violin plots revealed that the C1 neutrophil subset was primarily enriched in the M2 regulatory pattern, suggesting a unique transcriptional program (**Figure 7D**).

Regulon ranking based on regulatory specificity scores and activity variance further identified key TFs driving subpopulation-specific identity (**Figure 7E**). UMAP projections demonstrated the spatial distribution of each neutrophil subset in relation to their associated regulatory modules (**Figure 7F**). Functional enrichment of top regulons for each cluster revealed distinct transcriptional signatures: the C0 subpopulation was characterized by Hic1, Tcf7l2, Shox2, Zxdb, and Etv3; the C1 subpopulation by Stat2, Irf9, Irf8, Junb, and Irf7; the C2 subpopulation by Srebf2, Hes1, Bhlhe41, Atf3, and Usf2; and the C3 subpopulation by Fos, Rfx5, Stat3, Pbx1, and Stat1 (**Figure 7G**).

Comparative visualization via UMAP further highlighted subpopulation-specific TF expression landscapes (**Figures 7H, I**). Finally, the top five TFs defining the C1 (SLFN4^+^) cluster—Stat2, Irf9, Irf8, Junb, and Irf7—were visualized through both dimensional reduction and bar plot analysis, confirming their elevated and preferential expression in this inflammatory neutrophil subset (**Figures 7J, K**). These findings suggest that distinct TF circuits underlie neutrophil subset identity and function, with the C1 subpopulation governed by a STAT–IRF–JUNB regulatory axis, potentially driving its pro-inflammatory phenotype in TBI.

### 3.9 Clinical utility of a composite nursing prognostic model for TBI

In a prospective clinical cohort consisting of 30 patients diagnosed with moderate-to-severe traumatic brain injury (TBI), we constructed and applied a composite nursing prognostic model aimed at stratifying patient risk and guiding post-injury clinical management. The model integrated multiple prognostic dimensions, including initial neurological status (Glasgow Coma Scale), serum SLFN4 concentration as a molecular biomarker, systemic inflammatory indices (e.g., neutrophil-to-lymphocyte ratio), functional recovery metrics (Barthel Index), and the burden of in-hospital complications (e.g., pulmonary infection, seizure, electrolyte disturbance).

Patients were categorized into low-risk (n = 15), intermediate-risk (n = 6), and high-risk (n = 9) subgroups according to total composite scores. The average score across all patients was 12.8 ± 5.3, with a score range of 4.2 to 22.6. High-risk patients demonstrated significantly elevated serum SLFN4 levels (mean: 3.12 ng/mL, *P* < 0.001), higher mean number of complications during hospitalization (2.8 events per patient, *P* = 0.004), and substantially lower Barthel Index scores at discharge (mean: 38.6 ± 9.2, *P* < 0.001). Notably, 8 out of 9 patients in the high-risk group exhibited unfavorable neurological outcomes at 6-month follow-up, defined as Glasgow Outcome Scale–Extended (GOSE) ≤ 4.

#### 3.9.1 Predictive accuracy of the nursing prognostic score

Receiver operating characteristic (ROC) curve analysis confirmed the predictive value of the composite model. The area under the curve (AUC) for predicting poor functional outcome (GOSE ≤ 4 at 6 months) was 0.91 (95% CI: 0.82–0.99), indicating excellent discriminative capacity. A cutoff value of 15.2—determined by maximizing the Youden index—yielded a sensitivity of 88.9% and specificity of 86.7%. These findings suggest that the composite score may serve as a clinically useful and statistically robust tool for early identification of high-risk individuals who may benefit from enhanced nursing surveillance and tailored interventions.

#### 3.9.2 Stratified nursing interventions based on prognostic risk

To operationalize the prognostic model into clinical nursing practice, high-risk patients received individualized care plans incorporating intensified nursing strategies. These included:

##### 3.9.2.1 Early multidisciplinary intervention

Patients were enrolled in a multidisciplinary rehabilitation pathway involving neurology, physical therapy, nutrition, and psychiatric consultation within 72 hours of admission.

##### 3.9.2.2 Dynamic biomarker monitoring

Serial serum inflammatory markers including SLFN4, CRP, and IL-6 were monitored every 48 hours during the acute care phase.

##### 3.9.2.3 Structured follow-up nursing

A standardized post-discharge follow-up protocol was implemented for all high-risk patients. This included weekly telephone follow-ups for the first 3 months, home visit assessments at 1 and 3 months, and monthly nurse-led psychological support for patients and caregivers.

##### 3.9.2.4 Patient-centered education

Tailored education sessions on medication adherence, complication prevention, and signs of neurological deterioration were delivered to patients and their families prior to discharge, supplemented by printed home care manuals.

#### 3.9.3 Implications for precision nursing care

This composite model enables nurses to transition from reactive monitoring to proactive risk prediction, allowing for the deployment of resource-intensive care to those at highest risk. The incorporation of molecular biomarkers (e.g., SLFN4) and functional scales into a unified scoring framework exemplifies a precision nursing approach, aligning with current goals of personalized rehabilitation and nursing-sensitive outcomes in neurotrauma care.

### 3.10 Functional role of Slfn4 in regulating inflammation and migration after TBI

**Figure 8:**
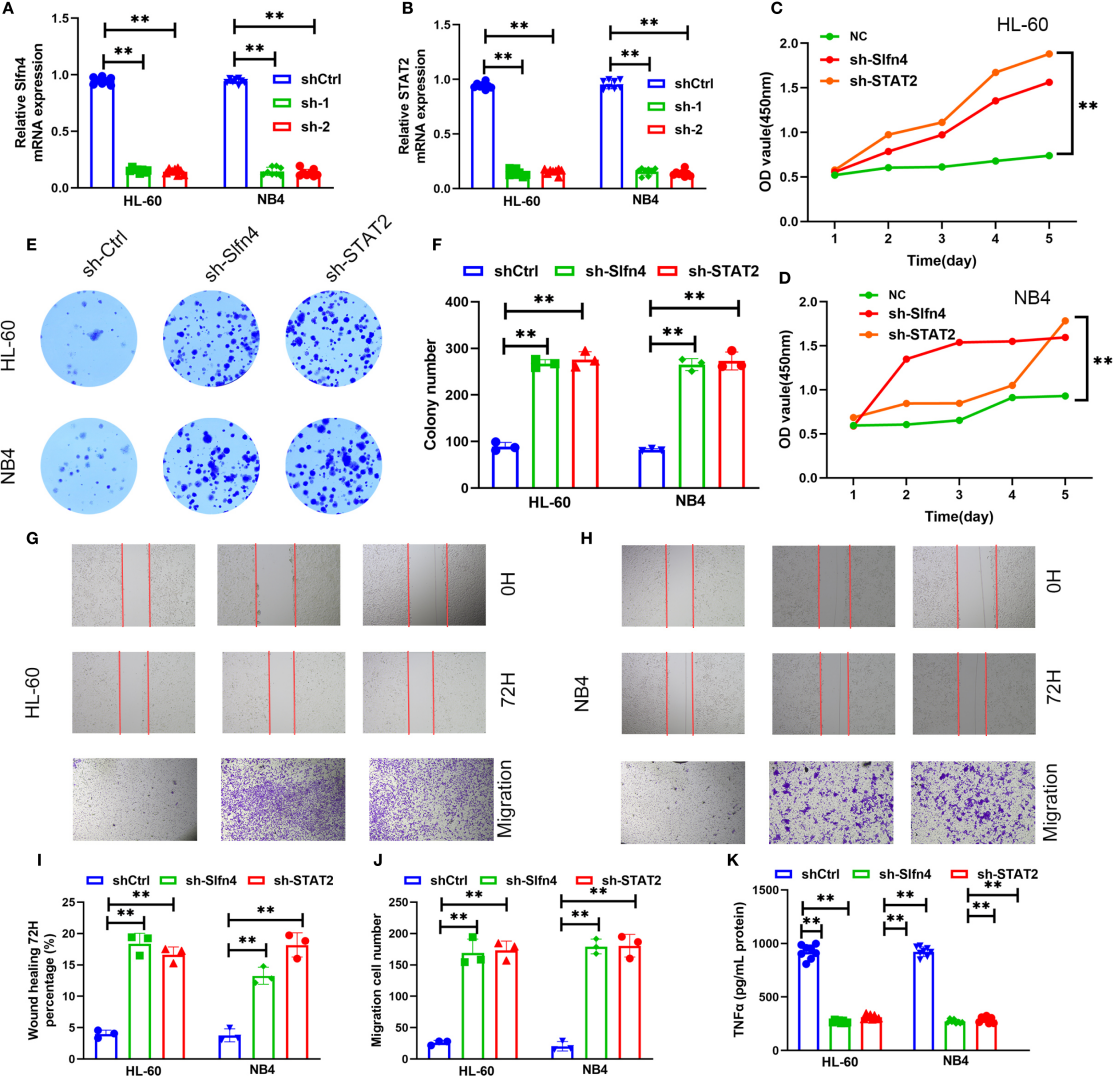
Functional assessment of Slfn4^+^ neutrophils in TBI. **(A, B)** Relative *Slfn4* and *STAT2* mRNA expression in HL-60 and NB4 cells after shRNA-mediated knockdown (sh-Ctrl, sh-1, sh-2). **(C, D)** Cell proliferation curves measured via CCK-8 assay over 5 days post-knockdown. **(E)** Representative colony formation assay images in HL-60 and NB4 cells. **(F)** Quantification of colony number. **(G, H)** Wound healing (0 h and 72 h) and Transwell migration assays across groups. **(I, J)** Quantitative analysis of wound closure percentage and number of migrated cells. **(K)** ELISA results of TNF-α and IL-6 levels in supernatants of HL-60, NB4 cells after LPS stimulation with or without *Slfn4*/*STAT2* knockdown.

To elucidate the functional significance of Slfn4^+^ neutrophils in the context of traumatic brain injury (TBI), we generated stable knockdown models targeting *Slfn4* and *STAT2* in neutrophil-like HL-60 and NB4 cell lines using two distinct short hairpin RNAs (shRNAs) per gene. Quantitative reverse transcription PCR confirmed efficient gene silencing in both cell lines (**Figures 8A, B**).

Cell proliferation analysis using the CCK-8 assay demonstrated that suppression of *Slfn4* or *STAT2* markedly enhanced proliferation over a 5-day period compared to control cells (**Figures 8C, D**). Similarly, colony formation assays indicated a substantial increase in clonogenic potential following gene knockdown (**Figures 8E, F**).

*In vitro* migration capacity was evaluated using wound healing and Transwell assays. Cells deficient in *Slfn4* or *STAT2* exhibited significantly accelerated wound closure and enhanced transmembrane migration (**Figures 8G–J**), suggesting that the Slfn4–STAT2 axis may exert a negative regulatory effect on neutrophil motility under neuroinflammatory conditions.

To assess inflammatory output, we stimulated the cells with lipopolysaccharide (LPS) and measured cytokine production in culture supernatants. ELISA quantification revealed a significant reduction in TNF-α secretion upon knockdown of either *Slfn4* or *STAT2* in both HL-60 and NB4 cells (**Figure 8K**), indicating that Slfn4^+^ neutrophils contribute to cytokine-driven inflammatory signaling following TBI.

Collectively, these results suggest that Slfn4^+^ neutrophils, via STAT2-dependent mechanisms, may play a dual role in modulating immune responses and cellular motility after brain injury, potentially acting as a regulatory brake on excessive inflammation and neutrophil infiltration.

## 4 Discussion

Traumatic brain injury (TBI) is increasingly recognized not only as a localized neuropathological event but as a systemic inflammatory disorder characterized by robust activation of innate immune responses—particularly those involving neutrophils (13). In this study, we employed a multi-layered omics strategy integrating single-cell transcriptomics, bulk RNA-seq, and proteomic profiling to dissect the immune complexity of the post-injury brain microenvironment (11, 12). Through this comprehensive approach, we identified a distinct neutrophil subpopulation marked by high *SLFN4* expression, which exhibited pronounced pro-inflammatory signatures and metabolic remodeling, and was found to be closely associated with clinical outcomes.

The current study presents a significant advancement in the integration of molecular immunology and nursing science by incorporating the novel biomarker SLFN4 into a composite prognostic framework for traumatic brain injury (TBI). Traditional nursing prognostic models primarily rely on clinical symptoms, functional scales, and patient-reported outcomes. However, these approaches often lack the sensitivity and specificity needed for early risk stratification, particularly in patients with complex systemic responses to neurotrauma.

By embedding SLFN4—a pro-inflammatory neutrophil marker identified through single-cell RNA sequencing—into a multifactorial follow-up scoring system, our model enables nursing professionals to dynamically evaluate disease progression and anticipate adverse outcomes with higher accuracy. This molecularly informed scoring strategy not only enhances the predictive power of conventional functional assessments (e.g., Barthel Index, GOSE), but also bridges the translational gap between laboratory research and front-line patient care.

From a nursing perspective, the implementation of this composite model allows for precision nursing interventions. High-risk individuals, as identified by the score, can be targeted for enhanced monitoring protocols, tailored caregiver education, early multidisciplinary rehabilitation, and structured follow-up. These strategies align closely with the principles of individualized nursing care, ensuring that interventions are both proactive and patient-centered.

Moreover, this study underscores the value of integrating omics data into the construction of nursing prognostic tools, offering a blueprint for future interdisciplinary research. It redefines the role of nursing professionals as not only caregivers but also active participants in data-driven decision-making, ultimately contributing to improved patient outcomes and quality of care in neurocritical settings.

The use of scRNA-seq enabled high-resolution delineation of neutrophil subsets and revealed a unique *SLFN4^+^* cluster with N1-like characteristics. These cells demonstrated elevated expression of inflammatory mediators and metabolic pathway enrichment, suggestive of a functionally primed state conducive to secondary injury propagation (36, 37). Concordant results from bulk transcriptome and proteomic datasets confirmed *SLFN4* as a robust biomarker within the acute neuroinflammatory response. Notably, serum *SLFN4* levels, prospectively measured in TBI patients, were strongly predictive of neurological prognosis and complication burden, underscoring its potential as a clinically actionable indicator of disease trajectory (38).

Functionally, *SLFN4* belongs to the Schlafen gene family, which is implicated in regulating interferon-mediated signaling, myeloid lineage maturation, and immune metabolic adaptation. Our data suggest that *SLFN4^+^* neutrophils may contribute to neuroinflammatory amplification through reactive oxygen species (ROS) generation, sustained pro-inflammatory cytokine secretion (e.g., TNF-α, IL-6), and persistent infiltration into injured tissue (39). These properties position the *SLFN4^+^* subpopulation as a promising candidate for targeted immunomodulation—potentially through interventions aimed at disrupting their metabolic or transcriptional programming (40).

Importantly, the clinical value of SLFN4 expression as a circulating biomarker has strong implications for precision nursing care and individualized risk assessment. In modern neurocritical care, timely identification of high-risk patients who are prone to exacerbated secondary injury or poor recovery remains a major challenge for frontline nurses and interdisciplinary teams. SLFN4^+^ neutrophil enrichment in peripheral blood may serve as an early warning signal, guiding nursing professionals to implement more intensive monitoring, enhanced neurological evaluation protocols, and timely coordination with neuroimmunology and rehabilitation teams (41).

Moreover, our longitudinal nursing follow-up data indicated that certain TBI patients experience sustained post-acute inflammation and delayed neurocognitive improvement, which may be partially attributed to prolonged activity of *SLFN4^+^* neutrophils. This maladaptive response highlights the need for strategies that can modulate neutrophil resolution dynamics in the subacute and chronic phases of TBI (42). From a nursing perspective, SLFN4-based immune profiling also enriches our understanding of inflammation-associated complications, such as delirium, autonomic dysfunction, or systemic immune suppression after TBI. Incorporating SLFN4 assessments into nursing risk stratification models, especially in the early post-injury phase, may facilitate decision-making regarding step-down care, frequency of neurochecks, and resource allocation. Moreover, as the importance of nurse-led follow-up clinics and post-discharge care grows, having measurable biomarkers like SLFN4 enhances longitudinal tracking of recovery trajectories and helps tailor psychosocial and rehabilitative interventions.

Despite the translational promise of our findings, several limitations should be acknowledged. The temporal dynamics of *SLFN4* expression during the different stages of TBI remain undefined, and our current analysis primarily centers on neutrophils without fully capturing their interactions with other immune or stromal cell types. Further studies employing spatial transcriptomics, lineage tracing, and targeted gene manipulation *in vivo* will be required to clarify the causal role of *SLFN4* and evaluate its potential as a therapeutic target in broader inflammatory neurological contexts (15). In addition, this study highlights the importance of interdisciplinary collaboration—bridging molecular immunology, translational medicine, and clinical nursing sciences—to develop integrative care strategies. Our findings call for further research into whether targeted modulation of SLFN4^+^ neutrophils (e.g., via anti-inflammatory agents or metabolic interventions) can alleviate secondary injury and improve functional outcomes, particularly from a nursing-sensitive outcomes perspective such as Glasgow Outcome Scale-Extended (GOSE), Barthel Index, and quality-of-life indicators.

In summary, our study identifies *SLFN4^+^* neutrophils as a mechanistically relevant, inflammation-associated subset defined by integrated multi-omics analysis. This population may serve as both a prognostic biomarker and a potential target for precision immunotherapy in TBI. These findings not only deepen our understanding of neuroimmune regulation but also illustrate the translational potential of multi-omics-guided approaches in advancing personalized care for neurotrauma patients.

## 5 Conclusions

This study identified SLFN4^+^ neutrophils as a distinct pro-inflammatory subset associated with poor prognosis in patients with traumatic brain injury (TBI). Through integrated multi-omics and clinical validation, SLFN4 was shown to serve as a potential biomarker for immune activation and neurological outcomes. Importantly, SLFN4 levels may facilitate early risk assessment and individualized care planning in neurocritical settings. These findings offer mechanistic insights into TBI-associated inflammation and provide a foundation for precision nursing strategies focused on immune monitoring and patient-centered outcome improvement.

## Data Availability

The datasets presented in this study can be found in online repositories. The names of the repository/repositories and accession number(s) can be found in the article/**Supplementary Material**.

## Supplementary material

Supplementary Figure 1 Flow chart of follow-up.
