# Supplementary material for: Single-cell multi-omics and nursing follow-up prognostic modeling reveal SLFN4-mediated neutrophil dysregulation in traumatic brain injury
Source: Front Immunol. 2025 Sep 23;16:1669800. doi: 10.3389/fimmu.2025.1669800 (PMC12500558; doi:10.3389/fimmu.2025.1669800)
Supplement: Supplementary file 1 [file Table1.docx]

# shRNA and qRT-PCR Primer Sequences

## shRNA Sequences

| **Target Gene** | **Target Sequence (19-21 nt)** | **TRC Clone ID** | **Strand** | **Vector Position** |
| --- | --- | --- | --- | --- |
| **Slfn4** | GCCAAGATCCTGAAGAAGATA | TRCN0000076353 | Forward | 5'-CCGG-[sequence]... |
|  | GCCTGTTCAAGTACCTGGAAA | TRCN0000076354 | Forward |  |
|  | GCTGGATGAACTCATTGAGAA | TRCN0000076355 | Forward |  |
| **STAT2** | GCACCTAAACTGCTCATCAAA | TRCN0000073201 | Forward | 5'-CCGG-[sequence]... |
|  | CCTGGAGATACAGTGTCATAT | TRCN0000073202 | Forward |  |
|  | GCTGCTACTTCAGCAACAGAA | TRCN0000073203 | Forward |  |

## qRT-PCR Primer Sequences

| Gene | Forward (5′→3′) | Reverse (5′→3′) |
| --- | --- | --- |
| Slfn4 | CAGCAGCTTCCAGTGATCCA | AGCCTTCTTGCTGTGGAGTT |
| STAT2 | CGAGACTGTGCCTGCTACAA | TTCCATTCCTGCTGTTGCTG |
| GAPDH | GAAGGTGAAGGTCGGAGTCA | GAAGATGGTGATGGGATTTC |
