# Supplementary figures and images for: Single-cell multi-omics and nursing follow-up prognostic modeling reveal SLFN4-mediated neutrophil dysregulation in traumatic brain injury
Source: Front Immunol. 2025 Sep 23;16:1669800. doi: 10.3389/fimmu.2025.1669800 (PMC12500558; doi:10.3389/fimmu.2025.1669800)

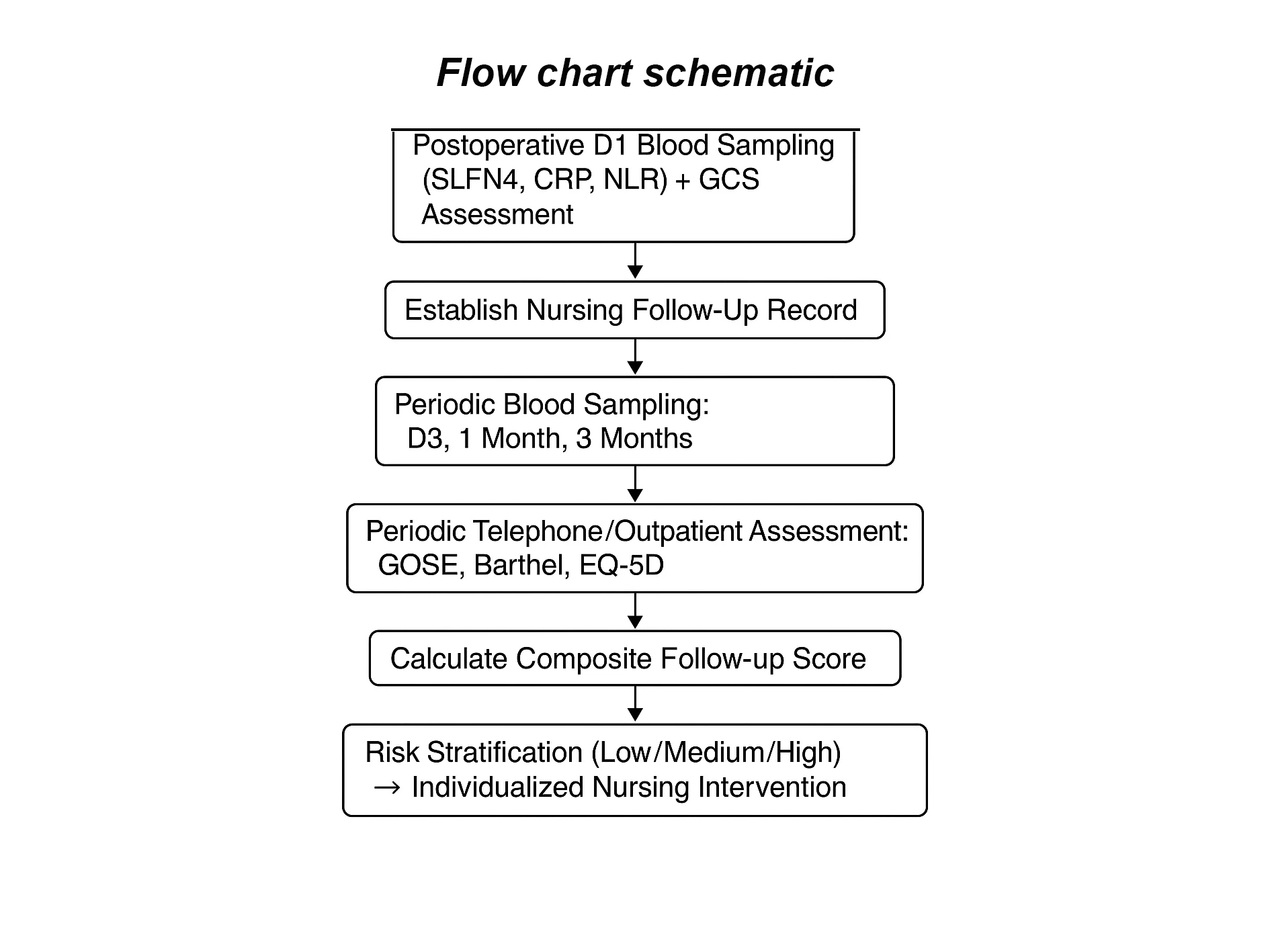

Supplement: Supplementary Figure 1 — Flow chart of follow-up. [file Image1.jpeg]
